# Cardiovascular-Renal-Hepatic-Metabolic Syndrome: Interlinked Pathophysiology and Integrated Management Approach

**DOI:** 10.7759/cureus.85813

**Published:** 2025-06-11

**Authors:** Carlos Alejandro Arragan Lezama, Julio Joel Jaramillo Ramos, David Alejandro Armas Eguizábal, Ximena Sofía Solares Ovando, Julio Cesar Minera Villagrán, Cinthya Jhoana Males Caiza

**Affiliations:** 1 General Practice, Universidad Anáhuac Xalapa, Veracruz, MEX; 2 Internal Medicine, Hospital Santo Tomás, Panama City, PAN; 3 Internal Medicine, Centro Médico Guatemala, Guatemala, GTM; 4 Internal Medicine, Centro Médico Militar, Guatemala, GTM; 5 Oncology, Instituto Guatemalteco de Seguridad Social, Guatemala, GTM; 6 General Practice, Universidad Nacional de Chimborazo, Otavalo, ECU

**Keywords:** advanced therapies, cardiovascular renal hepatic metabolic syndrome, crhm syndrome, emerging therapies, integrated management approach

## Abstract

Interdisciplinary research has revealed that heart, kidney, liver, and metabolic diseases share interlinked pathological mechanisms, including inflammation, oxidative stress, insulin resistance, and endothelial health issues. However, integrated care for these diseases is challenging due to different symptoms, diagnostic rules, and treatment options. The review aims to study the common mechanisms behind cardiovascular-renal-hepatic-metabolic syndrome and explore ways to coordinate management. When these systems work poorly together, damage can form a vicious cycle, increasing hypertension, chronic kidney disease, non-alcoholic fatty liver disease, and metabolic syndrome. Changes in medicine, daily lifestyle, and drugs can interrupt the pathological cascade. However, clinical practice is hindered by patient presentations, lack of teamwork across healthcare specialties, and incomplete therapy plans. Gut-organ axis dysfunction and inflammation play a significant role in multiple organ diseases, and prompt risk assessment and early intervention can prevent heart damage.

## Introduction and background

Non-communicable diseases, such as cardiovascular, renal, hepatic, and metabolic (CRHM) syndrome, are on the rise across the world, which leads to a situation in which diseases once treated separately are now recognized to be related and managed as a group, since many molecular mechanisms, inflammation throughout the body, and issues with hormone regulation are involved in all of them [1,2]. The cardiovascular, renal, hepatic, and metabolic systems are interrelated because the heart, kidneys, and liver collaborate to maintain homeostasis through blood filtration, metabolism, and hormonal regulation [3]. The heart is responsible for pumping blood with oxygen, the kidneys for filtering the blood, and the liver works to process foods and make necessary proteins and enzymes. Cardiac and renal functions interact by using neurohormonal signals [4]. Factors involving blood circulation, such as cardiac output, play a role in the blood perfusion and filtration in the kidney, which impacts the functioning of metabolic processes linked to glucose and lipids [5].

The main features of this syndrome are the overactivation of the renin-angiotensin-aldosterone system (RAAS). RAAS is characterized by an overactive sympathetic nervous system (SNS), insulin resistance, cell damage due to oxidative stress, and low-grade inflammation [6]. These pathways lead to heart failure, chronic kidney disease (CKD), non-alcoholic fatty liver disease (NAFLD), and diabetes, and when present together, they increase the overall health impacts [7]. CRHM syndrome must prompt health professionals to manage patients using multidisciplinary instead of organ-specific approaches [8]. If different parts of clinical care are handled separately, experts may ignore the combined issues and increased risks when several organs are affected [9]. So far, research prefers combining strategies that manage insulin resistance, inflammation, and fibrosis, along with efforts to control heart disease risk, maintain kidney function, protect the liver, and manage metabolism [10].

Chronic non-communicable diseases, such as cardiovascular disease, chronic kidney disease, non-alcoholic fatty liver disease, and metabolic syndrome, are a global epidemic. These disorders are often managed as isolated pathologies, but emerging clinical evidence supports a complex, multiorgan syndrome with shared etiological roots. However, there is a critical gap in clinical practice and research regarding the unified understanding and management of the cardiovascular-renal-hepatic-metabolic (CRHM) axis. Fragmented care, organ-specific therapeutic approaches, and a lack of integrated disease models often lead to suboptimal outcomes and the progression of organ dysfunction.

This review aims to highlight the anatomical, physiological, and molecular interdependence of these systems, emphasizing the importance of early, systems-based identification of risk, biomarker-driven diagnosis, and integrated therapeutic strategies. This research is intended to suggest a new way for clinicians, researchers, and policymakers to manage diseases by emphasizing a careful and integrated plan rather than just reacting to underlying disease conditions. Knowing about CRHM syndrome helps us treat chronic illness better, since it targets key problems that cause illness across other organs of the body.

## Review

Methodology

This literature review explores the underlying mechanisms and integrated management approach of cardiovascular-renal-hepatic-metabolic (CRHM) syndrome. A structured search of English-language, peer-reviewed articles was performed using databases including PubMed, ScienceDirect, and Google Scholar. The major search terms included Cardiovascular Renal Hepatic Metabolic Syndrome, CRHM syndrome, Integrated Management Approach, and advanced therapies using Boolean operators (AND, OR) to refine results. Articles were selected based on relevance and recent publication (preferably within the last five years).

Interlinked pathophysiology

The cardiovascular-renal-hepatic-metabolic (CRHM) syndrome occurs as a result of hemodynamic disorders, abnormal metabolic functions, stimulation of neurohormonal activity, and widespread inflammation. Such exchanges establish several ways in which organs communicate to create an ongoing disease.

Heart-Kidney Axis

Amabile and Geirsson concluded that heart failure occurs when the body holds on to fluids, causing blood pressure to rise and the heart to work harder. This reduced pumping leads to lower blood flow in the kidneys, causing extra fluid accumulation, electrolyte disturbances, and uremia, which intensifies heart problems and leads to cardiorenal syndrome [11]. Scurt et al. indicated that neurohormonal factors, such as RAAS, SNS, and anti-diuretic hormone (ADH), cause blood vessels to narrow, causing more sodium and water to be held onto and increasing pressure on the pumping heart [12]. However, atrial natriuretic peptide (ANP) and B-type natriuretic peptide (BNP) play a vital counteracting role in counteracting RAAS overactivation. Owing to increased blood volume and pressure, the heart released ANP, which increased sodium excretion by the kidney and increased fluid loss through diuresis. These counter-regulatory hormones reduce blood volume to lower blood pressure and alleviate the strain on the heart. Therefore, ANP and BNP play a protective compensatory mechanism against fluid overload to counterbalance the effect of other hormones that contribute to fluid retention [13]. Hence, ANP and BNP hormones serve as endogenous hormones that offer potential therapeutic targets for treating cardiovascular and renal diseases. 

Metabolic-Kidney Axis

Lv et al. indicated that insulin resistance, a part of metabolic syndrome, causes kidneys to work harder, leading to larger glomeruli and scarring. Hyperglycemia promotes the appearance of advanced glycation end products (AGEs), causing oxidative stress and nephropathy [13]. Ruiz-García et al.'s findings show that obesity increases pressure inside the kidneys and sodium uptake, increasing blood pressure and the risk of chronic kidney disease. Fat accumulation outside storage sites and inflammation cause progressive inner tubule damage, interstitial damage, and fibrosis [14].

Metabolic-Cardiac Axis

Obesity increases heart pressure, leading to left ventricular hypertrophy and concentric remodeling. Mikolasevic et al. indicated that inflammatory proteins from fat, called adipokines, interact with pro-inflammatory cytokines, causing stiffer arteries and increased cardiovascular risk [15]. Li et al. found out that metabolic syndrome disrupts the heart's energy use, causing inefficiency and diastolic issues. Fatty acid oxidation pathway disruption also exacerbates and dysregulates the axis [16].

Key molecular and hormonal mediators

The renin-angiotensin-aldosterone system (RAAS) plays a central role by promoting vasoconstriction, fluid retention, and fibrosis. Overactivation contributes to both hypertension and organ remodeling. The sympathetic nervous system (SNS), when chronically activated, causes tachycardia and vasoconstriction and contributes to the progression of both cardiac and renal disease [16]. Mottillo et al. showed that inflammatory cytokines, such as tumor necrosis factor alpha (TNF-α), IL-6, and CRP, amplify systemic inflammation, contributing to endothelial dysfunction, insulin resistance, and hepatic and myocardial injury. Adipokines released from adipose tissue also modulate organ function [17]. Nasrallah et al. found out that leptin increases sympathetic activity and vascular stiffness, while adiponectin, typically anti-inflammatory and vasoprotective, is decreased in obesity and metabolic syndrome. Oxidative stress resulting from mitochondrial dysfunction leads to DNA damage, lipid peroxidation, and cellular apoptosis in multiple organs, perpetuating systemic dysfunction [18]. According to Mutruc et al., in the kidneys, sodium-glucose cotransporter 2 (SGLT2) overactivity contributes to hyperglycemia, sodium retention, and glomerular hyperfiltration, further promoting renal injury and hypertension [19].

Vicious Cycle of Dysfunction

In cardiorenal syndrome, types 1 and 2, acute or chronic heart failure results in kidney injury due to low perfusion and venous congestion. Conversely, Sobhy et al. found out that in types 3 and 4, primary renal dysfunction impairs cardiac function via fluid overload, electrolyte disturbances, anemia, and increased inflammatory burden. Ultimately, this cyclical deterioration of interconnected systems results in compounded organ failure, reduced quality of life, frequent hospitalizations, and increased mortality, especially when hepatic dysfunction is also present [20].

Integrated management approach

*Cardiovascular Outcomes (Major Adverse Cardiovascular Event (MACE), Heart Failure* (*HF), Cardiovascular​​​​​​​* (*CV) Death)*

Among pharmacological therapies evaluated for CRHM management, SGLT2 inhibitors have consistently demonstrated the most robust and broad cardiovascular benefits, particularly in patients with type 2 diabetes mellitus (T2DM) and chronic kidney disease (CKD). Theodorakis and Nikolaou highlight that SGLT2 inhibitors, such as dapagliflozin and empagliflozin, significantly reduce hospitalizations for heart failure (HF), slow progression of renal disease, and lower the risk of major adverse cardiovascular events (MACE) [21]. According to Mutruc et al., SGLT2 inhibitors promoting osmotic diuresis, reducing preload and afterload, and improving endothelial function provide a strong protective effect against volume overload and cardiac stress, making them ideal for HF prevention [19]. Glucagon-like peptide-1 receptor agonist (GLP-1RAs) are better than other agents in decreasing the chances of atherosclerotic cardiovascular disease (ASCVD). Vora et al. deduce that, unlike SGLT2 inhibitors, GLP-1RAs don’t help with heart failure admissions or protect the kidneys. Still, they may help a person lose weight, control their blood pressure, and manage their blood sugar, all of which could benefit heart health over time [22]. Yamada et al. highlighted that using both types of drugs at the same time may be helpful because SGLT2 inhibitors help the heart, and GLP-1 receptor agonists aid weight loss and blood sugar control. When used in patients with several CRHM problems, this approach helps prevent heart failure and might reduce the risk of death from heart disease [23].

Another important new therapy that helps the heart is finerenone, a non-steroidal mineralocorticoid receptor antagonist (MRA). Filippatos et al. prove that finerenone lowers the chances of heart failure and cardiovascular death in patients with T2DM and CKD. Unlike older MRAs, finerenone carries a reduced risk of hyperkalemia, so it can be used safely for longer in people with heart and kidney problems. These anti-inflammatory and antifibrotic effects play a major role in lowering myocardial and renal fibrosis, which, in turn, strongly impacts how cardiovascular function improves over time [24]. Singh and Singh concluded that bempedoic acid is a treatment option for patients with high-risk heart disease (ASCVD) when statins are ineffective. It reduces low-density lipoprotein (LDL) cholesterol and reduces major cardiac events in high-risk patients. Although it doesn't directly reduce heart failure, its use in atherosclerotic event prevention makes it useful in ASCVD care [25].

According to Li et al., ACE inhibitors and angiotensin receptor blockers (ARBs) are the main treatments for protecting the heart in patients with CRHM. ACEi can control blood pressure, proteinuria, and cardiovascular events, especially in hypertensive and CKD patients [26]. In accordance with Chupradit et al.'s findings, ARBs also provide similar benefits but are less effective in reducing the overall risk of death. They adjust the renin-angiotensin-aldosterone system to avoid heart and vessel changes [27]. Bellucci et al. showed that colesevelam is a supplementary therapy that helps fight cardiovascular disease by lowering cholesterol and inflammatory CRP levels, particularly for type 2 diabetes patients. New treatments, such as phosphodiesterase-5 (PDE5) inhibitors like sildenafil, have improved cardiovascular function in diabetics by improving blood vessel lining and decreasing inflammation [28]. Tacke and Weiskirchen concluded that hydrogen sulfide (H₂S), often studied only in animals, could help neutralize oxidative stress and restore redox balance, providing new ways to protect the circulatory system [29]. SGLT2 inhibitors offer cardiovascular protection, particularly for heart failure and kidney issues. GLP-1RAs are particularly beneficial for atherosclerotic heart disease, and their combination with SGLT2i improves heart and metabolic health [29]. These medicines are particularly beneficial for those with CKD who cannot take statins. Treatment choices should be based on the main disease and any unusual risk factors.

*Renal Outcomes (Chronic Kidney Disease*​​​​​​​ *(CKD) Progression, Renal Events)*

According to Theodorakis and Nikolaou, the choice of drug for renal protection in CRHM depends on the type of drug used, as some drugs directly protect the kidneys while others control sugar, fats, or blood pressure [21]. Mutruc et al. concluded that SGLT2 inhibitors are the foundation of kidney protection in patients with diabetes and CKD, helping to reduce albuminuria, lower estimated glomerular filtration rate (eGFR) drops, and prevent end-stage kidney disease (ESKD). Dapagliflozin and empagliflozin prevent persistent kidney function drop, renal replacement therapy, and renal or cardiovascular death in patients with or without diabetes. These medications reduce glomerular pressure, decrease glucose absorption, reduce sodium, and ease inflammation [19]. Nissen et al. indicated that GLP-1 receptor agonists (GLP-1RAs) slow kidney decline by lowering macroalbuminuria and blood pressure. They can affect renal function but do not prevent ESKD as much as SGLT2 inhibitors. Improved blood sugar control, weight loss, and better circulation indirectly save kidneys over time [30].

It blocks the action of mineralocorticoid receptors and is an effective agent for guarding the kidneys in people with diabetes and CKD. It was shown in Chupradit et al.'s trials that finerenone can help treat albuminuria and cut the chances of kidney failure or issues with the heart [27]. Alcocer et al. concluded that unlike typical steroidal MRAs, finerenone is safer and causes fewer issues with hyperkalemia, which is very important for continuing renal treatment. Directly fighting inflammation and excessive scar formation helps prevent glomerular sclerosis and fibrosis in the interstitial tissues, two major problems in kidney disease progression [31]. ACEi and ARBs remain as key renal protective treatments in patients who are hypertensive and have proteinuria kidney disease. Alshahrani indicated that their use decreases pressure in the glomerular capillaries, reduces protein in the urine, and delays the decline of renal function [32]. Kaul et al. concluded that they are most helpful during the early and moderate stages of diabetic nephropathy, and following recommended guidelines for CKD include them. Even though they are not as strong as recent drugs, they are important parts of treatment combinations [33].

Cheng et al. suggest that bempedoic acid may help reduce renal risks, although these effects are seen indirectly. In the CLEAR Outcomes trial, bempedoic acid showed not only cardiovascular benefits but also a modest preservation of eGFR and reduction in inflammation. This suggests potential utility in patients with CKD and statin intolerance, although renal-specific trials are still needed for definitive conclusions [34]. Esan et al. found that other therapies like colesevelam and PDE5 inhibitors show limited but notable renal benefits. Colesevelam, through bile acid sequestration and lipid-lowering effects, may reduce systemic inflammation and slow CKD progression, though its renal benefits are secondary and less robust [35]. Li et al. showed that PDE5 inhibitors, such as sildenafil, demonstrate renoprotective potential via vasodilation and reduction in oxidative stress, but their use in renal disease remains largely experimental [26]. Barbagallo et al. have shown that experimental therapies targeting novel pathways, such as hydrogen sulfide (H₂S) donors, have shown promise in preclinical models by improving renal oxygenation and mitochondrial function and reducing fibrosis. However, their clinical relevance remains speculative until validated in large-scale human trials [36].

According to Singh and Singh, SGLT2 inhibitors provide the most robust and consistent renoprotective benefits across CRHM populations, significantly altering the course of CKD progression [25]. Filippatos et al. indicated that finerenone offers targeted antifibrotic effects and is especially valuable in diabetic nephropathy. While GLP-1RAs provide modest renal benefits, they are better suited for cardiovascular and metabolic risk control [24]. Alshahrani indicated that ACEi/ARBs are vital for proteinuria and hypertensive CKD and are most often used as the first therapy [32].

*Liver Health (Non-alcoholic Fatty Liver Disease*​​​​​​​ *(NAFLD), Metabolic-Associated Liver Disease​​​​​​​* *(MASLD), Hepatic Steatosis)*

Scammahorn et al. concluded that non-alcoholic fatty liver disease (NAFLD), metabolic-associated liver disease (MASLD), and hepatic steatosis are conditions that require proper liver health for treatment. However, these conditions are often overlooked due to high rates of NAFLD and non-alcoholic steatohepatitis (NASH) in patients with metabolic syndrome, T2DM, and CVD. Pharmacological approaches in CRHM impact not only the heart, kidneys, and metabolism but also the liver [37].

Among all agents used in CRHM care, GLP-1 receptor agonists (GLP-1RAs) reliably help the most with results on the liver. As noted by Theodorakis and Nikolaou and Singh and Singh, agents such as liraglutide and semaglutide reduce hepatic steatosis and improve liver enzyme profiles [21,25]. Tacke and Weiskirchen have shown reductions in liver fat content and histological improvements in NASH with GLP-1RAs. These benefits are largely attributed to weight loss, improved insulin sensitivity, and anti-inflammatory effects [29]. GLP-1RAs are among the most promising treatments for patients with concurrent NAFLD and T2DM, and several are in phase III trials for NASH-specific indications.

SGLT2 inhibitors are primarily reno-cardioprotective and also exert favorable hepatic effects. Agents like dapagliflozin and empagliflozin reduce hepatic fat content and improve liver enzymes such as ALT and AST. Their benefits, though generally more modest than GLP-1RAs, arise from improvements in glycemic control, visceral fat reduction, and insulin sensitivity [21]. Nissen et al. show reduced NAFLD progression and early signs of fibrosis attenuation, but large-scale histological studies are still ongoing. Thus, SGLT2 inhibitors are considered supportive agents in NAFLD/NASH management, particularly in patients with coexisting T2DM or CKD [30].

Finerenone** **is a nonsteroidal MRA that has shown emerging evidence of liver benefit, particularly due to its antifibrotic and anti-inflammatory properties. While Alcocer et al. primarily studied in the context of renal and cardiovascular disease, preclinical data and post-hoc analyses suggest finerenone may reduce hepatic inflammation and fibrosis, which are central to NASH progression [31]. This is supported by Chupradit et al., showing modulation of fibrotic markers and inflammatory gene expression. However, dedicated trials in liver disease populations are still needed to confirm these effects [27]. Statins, including atorvastatin and rosuvastatin, are not traditionally considered hepatoprotective due to early concerns about hepatotoxicity. However, Kaul et al. support their safety and potential benefits in patients with NAFLD/NASH. Statins improve liver enzyme levels and reduce cardiovascular risk in patients with metabolic liver disease. Their pleiotropic effects--anti-inflammatory, endothelial-protective, and antifibrotic--contribute indirectly to liver health. They are now recommended for dyslipidemia patients with NAFLD, even in the presence of mildly elevated transaminases [33].

Bempedoic acid is an ATP-citrate lyase inhibitor used in statin-intolerant patients and offers a novel mechanism of lipid-lowering. Though data on its hepatic effects are limited, Nissen et al. showed no hepatotoxicity and modest reductions in liver enzymes, suggesting a safe profile. Its role in NAFLD/NASH remains undefined, though it could be a useful alternative for lipid management in hepatic-compromised individuals [30]. On the other hand, colesevelam, a bile acid sequestrant, shows mixed hepatic effects. While it can reduce LDL-C and improve glycemic control, it may increase hepatic triglyceride synthesis in some cases. Although Esan et al. suggest that brain-specific angiogenesis inhibitor-1 (BAI1) could benefit insulin resistance in the liver, the use of this drug is still very limited for people with non-alcoholic fatty liver disease/non-alcoholic steatohepatitis (NAFLD/NASH) [35].

Sildenafil, tirapazamine, and H₂S donors have been found to improve liver circulation, maintain healthy blood vessels, and reduce fibrosis in animals. Yamada et al. suggested that GLP-1 receptor agonists are currently the best option for improving the liver in CRHM, providing histologic and metabolic benefits for people with NAFLD/NASH. SGLT2 inhibitors also help the liver by reducing weight and increasing insulin sensitivity [23]. According to Chupradit et al., finerenone and statins are promising for patients with heart and metabolic illnesses [27]. Singh and Singh further emphasize that bempedoic acid, colesevelam, and PDE5 inhibitors are effective, but more research is needed to prove their benefits in the liver. Individualizing care for liver problems is crucial in CRHM [25].

Metabolic Control (Glycemic, Lipid, Weight Outcomes)

At the heart of CRHM syndrome, problems with metabolism cause and worsen issues in the heart, kidneys, and liver. It is therefore necessary to manage metabolic aspects, including controlling sugar, insulin resistance, blood fats, body fat stores, and inflammation. The medicines used in CRHM have diverse ways of benefiting metabolic health. Some directly target metabolism, while others help indirectly through effects on other organs.

CRHM patients benefit from the powerful effects of GLP-1 receptor agonists on metabolic improvement. Bellucci et al. concluded that all three drugs--liraglutide, semaglutide, and dulaglutide--have been demonstrated to lower hemoglobin A1c, keep weight down, increase insulin sensitivity, and decrease generalized inflammation [28]. Alshahrani observes that GLP-1RAs work better than other diabetes drugs in lowering body weight and maintaining healthy blood sugar levels. They contribute to lower post-meal fat levels and make modest improvements in the body’s lipid profile [32]. These multiple benefits are especially important for people who have obesity complications, type 2 diabetes, and fatty liver disease-conditions often found together in CRHM. SGLT2 inhibitors support metabolic health, mostly by helping to control blood sugar and reduce excess weight. According to Filippatos et al., though the glucose-lowering action of SGLT2-like empagliflozin and dapagliflozin is not as strong as that of GLP-1RAs, they help the body get rid of calories through glycosuria and lower body weight and visible abdominal fat, too [24]. They also modestly improve insulin sensitivity and exert favorable effects on uric acid and blood pressure. Theodorakis and Nikolaou confirm consistent metabolic improvements, especially in patients with type 2 diabetes. Their benefits extend beyond glycemic parameters to metabolic flexibility and improved mitochondrial function, although they have limited effects on lipid parameters [21].

Finerenone is primarily used for its renal and cardiovascular effects and shows emerging metabolic benefits by modulating pro-inflammatory and pro-fibrotic pathways associated with insulin resistance and metabolic syndrome. Tacke and Weiskirchen suggest mild improvements in glycemic parameters and a reduction in markers of systemic inflammation. Though not a first-line metabolic therapy, finerenone may indirectly improve metabolic health in patients with overlapping CKD and diabetes [29]. Statins and bempedoic acid** **are not only metabolic drugs but also contribute significantly to metabolic health by improving lipid profiles, particularly LDL-C reduction, and reducing inflammation. Alcocer et al. indicated that despite concerns about slight increases in blood glucose levels, especially with high-potency statins, their overall benefit in reducing atherogenic risk and stabilizing metabolic status in CRHM patients is well-documented [31]. Nissen et al. concluded that bempedoic acid, a newer lipid-lowering agent, has been shown in the CLEAR Outcomes trial to reduce LDL-C by up to 20-25% without the glycemic concerns associated with statins, making it a favorable option in patients with diabetes or statin intolerance [30].

Colesevelam is a bile acid sequestrant that has dual benefits in improving lipid and glycemic control. It lowers LDL cholesterol and modestly reduces hemoglobin A1c in type 2 diabetes patients. Esan et al. report that it may also improve hepatic insulin sensitivity and gut hormone profiles, though gastrointestinal side effects and pill burden limit its widespread use [35]. PDE5 inhibitors (e.g., sildenafil) and hydrogen sulfide (H₂S) donors show potential metabolic benefits. Barbagallo et al. suggested that PDE5 inhibitors may enhance insulin sensitivity, endothelial function, and microvascular blood flow, particularly in obese or insulin-resistant individuals [36]. Scammahorn et al. indicated that H₂S donors, though still in preclinical stages, appear to improve mitochondrial efficiency and reduce inflammation, offering a novelty in metabolic restoration [37].

GLP-1 receptor agonists offer the most comprehensive and impactful metabolic improvements among therapies in CRHM, making them ideal for patients with obesity, type 2 diabetes, and fatty liver disease [28,32]. SGLT2 inhibitors provide additional benefits through glycosuria-induced weight loss and improved insulin sensitivity [21,24]. Finerenone plays a secondary role by fighting inflammation and fibrosis [29], with statins and bempedoic acid essential for controlling lipids [30,31]. It reduces blood sugar and cholesterol, though some people find it difficult to take. New types of drugs, such as PDE5 inhibitors and H₂S donors, show potential for future improvements in metabolic disorders [36,37]. The best way to manage CRHM is to target all parts of the metabolic axis to stop the progression of damage to the heart, kidneys, and liver.

Clinical efficacy and challenges

The effectiveness of different types of medicines used in CRHM management can be very different, and each drug tends to improve particular features of the condition. Mutruc et al. confirm that GLP-1 receptor agonists like semaglutide, liraglutide, and dulaglutide significantly decrease the risk of stroke and myocardial infarction [19]. Vora et al. also offer a little shielding to the kidneys by doing things like lowering albuminuria and slowing the decline in eGFR [22]. Apart from this, Yamada et al. concluded that GLP-1RAs contribute to substantial weight loss and drops in HbA1c scores from 1.0% to 1.5%, as well as improved insulin sensitivity [23]. Filippatos et al. indicated that they work well in the liver; semaglutide helped resolve 59% of cases of non-alcoholic steatohepatitis (NASH), with no worsening of scarring, as was observed in a large NASH study [24].

In contrast, SGLT2 inhibitors, such as empagliflozin, dapagliflozin, and canagliflozin, are most impactful in heart failure and renal outcomes. Li et al. concluded that cardiovascular efficacy is well supported by trials that demonstrate 25-38% reductions in heart failure hospitalizations and cardiovascular death, especially in patients with heart failure with reduced ejection fraction (HFrEF) [26]. Tacke and Weiskirchen indicated that their renal protection is the most robust among current drug classes, showing substantial delays in CKD progression, reduced albuminuria, and sustained eGFR benefits [29]. According to Nissen et al., hepatic outcomes are more modest, with some reduction in hepatic steatosis reported in smaller studies, though consistent histological improvements in NASH have not been observed. Metabolically, SGLT2 inhibitors achieve modest HbA1c reductions (0.5-1.0%) and support weight loss, though their effect is less pronounced than GLP-1RAs [30].

Chupradit et al. reported that finerenone, a non-steroidal mineralocorticoid receptor antagonist, shows moderate cardiovascular benefit, particularly in reducing heart failure outcomes in patients with chronic kidney disease and type 2 diabetes [27]. Alcocer et al. showed that renal benefits are significant, with consistent improvements in albuminuria and delayed CKD progression, establishing it as a critical therapy in diabetic kidney disease. While hepatic data remain limited, preclinical studies suggest antifibrotic effects. Metabolic outcomes with finerenone are largely neutral, offering no significant changes in weight or glycemic control [31].

Statins remain the cornerstone of cardiovascular risk reduction, with well-established efficacy in reducing MACE by 25-30%, as demonstrated in large trials by Alshahrani [32]. Kaul et al.'s study offers minor benefits, such as slowing early CKD progression and reducing proteinuria. In hepatic conditions, statins are safe and may modestly lower transaminase levels in NAFLD but do not reverse fibrosis or improve NASH histology [33]. Metabolically, statins improve lipid profiles but slightly increase the risk of new-onset diabetes, especially at high doses. Bempedoic acid, as demonstrated by Cheng et al., provides a moderate cardiovascular benefit (13% MACE reduction) in statin-intolerant patients but lacks significant renal or hepatic effects. Metabolically, it is neutral in terms of glucose metabolism and does not promote weight loss [34]. Esan et al. found out that colesevelam, though primarily a lipid- and glucose-lowering agent, has limited cardiovascular data and no renal outcomes. It modestly improves glycemic control (HbA1c reduction ~0.5%) and is useful as an adjunct in type 2 diabetes [35].

According to Chupradit et al., phosphodiesterase-5 (PDE5) inhibitors, such as sildenafil and tadalafil, have emerging cardiovascular roles in improving endothelial function and symptoms in pulmonary hypertension and heart failure with preserved ejection fraction (HFpEF), with early evidence suggesting possible renal and hepatic benefits [27]. Scammahorn et al. conclude that metabolically, they may improve insulin sensitivity, though large-scale data remain limited. Lastly, hydrogen sulfide (H₂S) donors demonstrate promising preclinical efficacy across all CRHM domains, including antifibrotic, anti-inflammatory, and insulin-sensitizing effects, but their clinical utility awaits validation in human trials [37].

Comprehensive review of current literature 

Table 1 explains the characteristics and findings of studies included in the review; it encompasses author, year of study, study design/population, objectives, interventional protocol, disease involved, outcomes measured, study findings, and overall conclusion.

**Table 1:** Studies included in review. CRHM: cardiorenal-hepatic-metabolic, SGLT2i: sodium-glucose cotransporter-2 inhibitor, GLP-1RA: glucagon-like peptide-1 receptor agonist, GIP: gastric inhibitory polypeptide, T2DM: type 2 diabetes mellitus, CVD: cardiovascular disease, HF: heart failure, CKD: chronic kidney disease, MASLD: metabolic dysfunction-associated steatotic liver disease, ASCVD: atherosclerotic cardiovascular disease, GI: gastrointestinal, CKM: cardiovascular-kidney-metabolic, RAAS: renin-angiotensin-aldosterone system, CaReMe: cardio-reno-metabolic, RCT: randomized controlled trial, MACE: major adverse cardiovascular events, CVOT: cardiovascular outcomes trial, SBP: systolic blood pressure, HHF: hospitalization for heart failure, HR: hazard ratio, PPSP: probiotics, prebiotics, synbiotics, and postbiotics, GDM: gestational diabetes mellitus, IR: insulin resistance, NAFLD: non-alcoholic fatty liver disease, FXR: farnesoid X receptor, MCJ: methylation-controlled J protein, RES: resveratrol, TNF-α: tumor necrosis factor alpha, IL-1α: interleukin 1 alpha, IL-1β: interleukin 1 beta, IL-6: interleukin 6, NF-κB: nuclear factor kappa B, PI3K: phosphoinositide 3-kinase, Akt: protein kinase B, mTOR: mammalian target of rapamycin, MDA: malondialdehyde, NO: nitric oxide, CAT: catalase, SOD: superoxide dismutase, GSH: glutathione, AST: aspartate aminotransferase, ALT: alanine aminotransferase, ACEi: angiotensin-converting enzyme inhibitor, ARB: angiotensin receptor blocker.

Author/year	Study design/population	Objectives	Interventional protocol/treatment	Disease involved	Outcomes measured	Study findings	Overall conclusion	Complications	Challenges
Theodorakis and Nikolaou [21]	Narrative review: Patients with overlapping cardiovascular, renal, hepatic, and metabolic conditions	To introduce and define the CRHM syndrome concept; To evaluate the integrated therapeutic potential of SGLT2 inhibitors, GLP-1RAs, and dual GIP/GLP-1RAs; To advocate for cross-specialty and unified management approaches	Clinical trials on: SGLT2 is (dapagliflozin, empagliflozin); GLP-1RAs (semaglutide); GIP/GLP-1RA (tirzepatide)	Obesity, type 2 diabetes mellitus (T2DM), atherosclerotic cardiovascular disease (CVD), heart failure (HF), chronic kidney disease (CKD), metabolic dysfunction-associated steatotic liver disease (MASLD), metabolic syndrome	Heart failure hospitalization and mortality - CKD progression - Liver health (MASLD markers) - Glycemic control - Weight loss - Risk reduction for T2DM - Atherosclerotic cardiovascular events	SGLT2 is improve HF and CKD outcomes, regardless of diabetes status - GLP-1RAs support weight loss and improve ASCVD and liver disease outcomes - Tirzepatide shows superior results in weight reduction (>20%), diabetes prevention, and MASLD improvement	These agents should be integrated across specialties as a unified treatment model for CRHM syndrome, moving beyond siloed, organ-based approaches toward precision cardiometabolic medicine	GI issues with GLP-1RAs)	Lack of guideline integration across specialties; Fragmented healthcare delivery models; Need for collaborative management frameworks and better physician awareness/training; Incomplete trial data for some organ combinations (especially liver outcomes)
Mutruc et al. [19]	Narrative review: Patients with overlapping cardiovascular, kidney, and metabolic disorders (e.g., heart failure, chronic kidney disease (CKD), diabetes, obesity, hypertension, metabolic syndrome)	To explain and contextualize the CKM (cardiovascular–kidney–metabolic) syndrome; To examine shared pathophysiology across the three systems; To present diagnostic and therapeutic approaches; To promote holistic, integrative clinical strategies	Lifestyle modification - Glycemic and lipid control - Cardio- and nephroprotective medications (e.g., RAAS inhibitors, SGLT2 inhibitors)	Cardiovascular, renal, and metabolic systems	Reduced mortality and morbidity; Slower CKD progression; Improved cardiovascular outcomes; Better glycemic/lipid control	CKM syndrome reflects interdependent deterioration between heart, kidney, and metabolic health; Common drivers include inflammation, oxidative stress, RAAS activation, insulin resistance; Unified approaches improve outcomes more than siloed treatment	CKM syndrome represents a modern, clinically relevant framework requiring integrated, multidisciplinary management to break the cycle of organ dysfunction and improve outcomes	Not reported	Lack of early diagnostic markers; Clinical inertia and specialty-based silos; Need for awareness, education, and collaboration across care teams
Vora et al. [22]	Review/Expert Commentary; Patients with coexisting cardiovascular, renal, and metabolic diseases	To explore shared mechanisms of CaReMe diseases and emphasize the need for integrated care approaches	Pharmacological (e.g., agents with cardio–reno–metabolic benefits), non-pharmacological interventions; integrated, team-based care models	Cardiovascular, Renal, and Metabolic (CaReMe) diseases	Patient outcomes (morbidity, mortality); adherence to guidelines; care efficiency	CaReMe diseases share common pathophysiology (e.g., insulin resistance, inflammation); integrated care improves early diagnosis, adherence, and patient outcomes	Holistic, interdisciplinary care models improve management of CaReMe diseases, reduce healthcare burden, and enhance patient experience	Not directly addressed; general risks of chronic disease management apply	Fragmented care systems, lack of interdisciplinary coordination, guideline non-adherence, resource inefficiencies
Yamada et al. [23]	Systematic review and network meta-analysis of 13 RCTs (N=32,949 patients with type 2 DM and CKD)	To compare the efficacy of SGLT-2 inhibitors vs GLP-1 RAs in reducing cardiovascular and renal events in type 2 DM with CKD	SGLT-2 inhibitors and GLP-1 receptor agonists (GLP-1 analogs and exendin-4 analogs)	Type 2 diabetes mellitus with chronic kidney disease (CKD)	Major adverse cardiovascular events (MACE), composite renal outcomes	GLP-1 analogs reduced MACE (RR 0.81), while exendin-4 analogs did not.	SGLT-2 inhibitors are more effective in reducing cardiovascular and renal events than GLP-1 RAs in type 2 DM with CKD.	Not reported	Limited by indirect comparison and heterogeneity in GLP-1 RA subclass effects (e.g., GLP-1 analogs vs exendin-4 analogs)
Singh and Singh [25]	Comprehensive opinion review with meta-analysis of five CVOTs and two RCTs	To evaluate the metabolic and cardiovascular benefits of GLP-1RA and SGLT-2I combination therapy in type 2 diabetes	Combination therapy: GLP-1 receptor agonists + SGLT-2 inhibitors	Type 2 diabetes mellitus	HbA1c, body weight, systolic blood pressure (SBP), MACE, heart failure hospitalization	HbA1c reduction was less than additive, body weight reduction nearly additive, and SBP reduction more than additive. CV benefits (MACE) were similar to monotherapy. Combination therapy showed greater benefit in reducing heart failure hospitalizations in pooled analysis.	Combination therapy provides enhanced metabolic and potential cardiovascular benefits, particularly for heart failure reduction.	Not reported	Comprehensive opinion review with meta-analysis of five CVOTs and two RCTs
Filippatos et al. [24]	Prespecified analysis of FIGARO-DKD RCT (N = 7352 patients with T2DM and albuminuric CKD)	To assess the effect of finerenone on heart failure (HF) outcomes in CKD and T2DM patients	Finerenone vs. placebo	Type 2 diabetes mellitus with chronic kidney disease (CKD)	New-onset HF, first HHF, CV death or HHF, total HHF, HF-related death or HHF	Finerenone significantly reduced: new-onset HF (HR 0.68), first HHF (HR 0.71), CV death or first HHF (HR 0.82), and total HHF (rate ratio 0.70). Benefits were consistent regardless of HF history.	Finerenone effectively reduces the risk of heart failure events and cardiovascular death in CKD + T2DM patients, independent of prior HF history.	Not reported	No direct evaluation of ejection fraction outcomes; HF subtypes not separately analyzed
Li et al. [26]	Narrative review (preclinical & clinical studies included)	To evaluate effects and mechanisms of probiotics, prebiotics, synbiotics, and postbiotics (PPSP) in managing metabolic diseases via modulation of gut microbiota	Use of PPSP to modulate gut microbiota in obesity, T2DM, GDM, etc.	Obesity, T2DM, gestational diabetes mellitus (GDM), other metabolic diseases	Changes in gut microbiota composition, microbial metabolites, intestinal barrier function, metabolic parameters	PPSP improve microbiota diversity, reduce inflammation, enhance intestinal integrity, and improve glycemic and lipid profiles	PPSP show promising potential in managing metabolic diseases by targeting gut microbiota, though optimal formulations and GDM effects need further study	Not reported	Standardization of PPSP formulations; limited evidence for GDM; variability in human trials
Bellucci et al. [28]	Narrative review	To examine the pathological role of gut dysbiosis in linking NAFLD and insulin resistance, and to explore novel therapeutic approaches	Probiotics, prebiotics, symbiotics, postbiotics, fecal microbiota transplant, Chinese herbal medicine, antibiotics, polyphenols, fasting diets, carbon nanoparticles, MCJ protein, hydrogen-rich water	NAFLD, insulin resistance (IR), gut dysbiosis	Gut permeability, inflammation, liver steatosis and fibrosis, IR parameters	Dysbiosis contributes to IR and NAFLD progression; various therapies showed promise in improving gut barrier integrity and reducing liver pathology	Dysbiosis contributes to IR and NAFLD progression, with various therapies promising in improving gut barrier integrity and reducing liver pathology.	Not reported	To examine the pathological role of gut dysbiosis in linking NAFLD and insulin resistance, and to explore novel therapeutic approaches
Tacke and Weiskirchen [29]	Narrative review (preclinical and clinical trial data)	To review recent therapeutic strategies targeting the bile acid receptor FXR for NAFLD and cholestatic/metabolic liver diseases	FXR agonists (steroidal and non-steroidal), pan-FXR vs. intestinal-selective activation, FXR antagonists	NAFLD, cholestatic and metabolic liver diseases	Lipid metabolism, hepatic inflammation, hepatocellular toxicity, pruritus, atherogenic profile	FXR activation improves liver metabolism but may cause side effects like pruritus, pro-atherogenic profiles; selective intestinal FXR targeting could mitigate these	FXR remains a promising drug target, but selective activation may offer therapeutic advantage over full FXR agonism	Pruritus, atherogenic lipid profiles, hepatotoxicity with unrestricted FXR activation	Balancing efficacy with tolerability; uncertainty about optimal FXR activation mode; need for more clinical data
Nissen et al. [30]	Randomized, double-blind, placebo-controlled trial; 4206 statin-intolerant patients (primary prevention)	To assess if bempedoic acid reduces major cardiovascular events in statin-intolerant patients without prior CVD	Oral bempedoic acid 180 mg daily vs. placebo	Cardiovascular disease prevention in statin-intolerant patients	Composite of CV death, MI, stroke, coronary revascularization; LDL-C and hs-CRP levels	Bempedoic acid significantly reduced primary endpoint (HR 0.70); LDL-C reduced by 21.3%; hs-CRP by 21.5%; reduced MI and CV death but not stroke or revascularization	Bempedoic acid is effective for reducing major CV events in high-risk statin-intolerant patients for primary prevention	Gout (2.6%), cholelithiasis (2.5%), ↑ serum creatinine, uric acid, liver enzymes	Monitoring required for metabolic and renal adverse events; not effective on all CV endpoints (e.g., stroke)
Chupradit et al. [27]	Narrative review of preclinical and clinical studies	To summarize the hepatoprotective and therapeutic effects of resveratrol focusing on anti-inflammatory and antioxidative activities	Resveratrol (RES) treatment from various sources (e.g., grapes, red wine) studied in liver disease models	Various liver diseases: alcoholic liver disease, hepatic steatosis, liver fibrosis, NAFLD, hepatocellular carcinoma, hepatic failure	Inflammatory cytokines (TNF-α, IL-1α, IL-1β, IL-6), NF-κB activity, PI3K/Akt/mTOR pathway, oxidative stress markers (MDA, NO, CAT, SOD, GSH), liver injury enzymes (AST, ALT)	RES reduced liver inflammation by downregulating pro-inflammatory cytokines and NF-κB, induced apoptosis via PI3K/Akt/mTOR inhibition, reduced oxidative stress markers, increased antioxidant enzymes, decreased liver injury markers	RES is a safe, natural antioxidant with anti-inflammatory, anti-apoptotic, and anti-fibrotic effects providing hepatoprotection and therapeutic benefits	No major complications reported; RES considered safe in reviewed studies	Need for more clinical trials to confirm efficacy and optimal dosing in humans
Alcocer et al. [31]	Review article: Critical appraisal of guidelines	Hypertensive patients, with/without cardiovascular comorbidities	To appraise and compare international guidelines on ACEis vs ARBs in hypertension management and cardio-renal protection	ACE inhibitors vs ARBs	Both ACEis and ARBs effectively lower blood pressure; ACEis preferred in some guidelines for CV comorbidities; ARBs used when ACEis not tolerated. Mechanistic differences include ACEi-induced bradykinin increase and nitric oxide stimulation, which may contribute to better CV and renal outcomes	ACEis and ARBs should be considered distinct drug classes within RAAS inhibitors; ACEis have broader cardio-renal benefits	ACEis are often first choice unless contraindicated; ARBs as alternatives; treatment should consider patient comorbidities	Not reported	Review article/Critical appraisal of guidelines
Alshahrani [32]	Review article; patients with chronic kidney disease (CKD)	To compare RAAS modulators—direct renin inhibitor, ACE inhibitors, and ARBs—in CKD management	Analysis of mechanisms, efficacy, and safety of Aliskiren, ACEis, and ARBs	Chronic kidney disease (CKD)	Blood pressure control, renal function, proteinuria reduction	RAAS modulators are first-line treatment; ACEis and ARBs reduce proteinuria and slow CKD progression; aliskiren less studied but effective	ACEis and ARBs remain cornerstone drugs; choice depends on patient presentation and drug tolerability	Hyperkalemia, renal function deterioration, hypotension noted with RAAS modulators	Lack of direct head-to-head trials; variability in patient responses; affordability and access issues
Kaul et al. [33]	Integrated analysis of 18 real-world clinical studies (five manuscripts + 13 abstracts); total 5824 patients with diabetic dyslipidemia; mean age 49.6–59.1 years; 22–42% female; treatment duration 12–58 weeks	To summarize effects of Saroglitazar on lipid and glycemic parameters in diabetic dyslipidemia patients in real-world clinical practice post-marketing authorization	Saroglitazar 4 mg once daily for at least 12 weeks (range 12 to 58 weeks)	Metabolic system: lipid metabolism, glycemic control, liver function	Diabetic dyslipidemia and NAFLD	Lipid parameters (triglycerides, total cholesterol, LDL-C, HDL-C, non-HDL cholesterol), glycosylated hemoglobin (HbA1c), alanine aminotransferase (ALT), fatty liver (FibroScan)	Mean reduction in triglycerides (45–62%), total cholesterol (17–26%), non-HDL cholesterol (21–36%), LDL cholesterol (11–27%), HbA1c (0.7–1.6%), increase in HDL cholesterol (up to 9%); Improvement in ALT and fatty liver scores; No significant changes in body weight; No significant adverse events reported	Not reported	No significant adverse events reported
Cheng et al. [34]	Review of existing literature; no primary population	To elucidate the role of curcumin in mitigating oxidative stress and alleviating lipid metabolism disorders, and to explore its therapeutic potential in metabolic diseases	Not applicable (review of multiple studies on curcumin)	Oxidative stress pathway, lipid metabolism	Lipid metabolism disorders including hyperlipidemia, NAFLD, atherosclerosis, obesity, diabetes mellitus	Measures discussed include reduction in reactive oxygen species (ROS), fat deposition, fatty acid uptake, insulin sensitivity	Curcumin reduces oxidative stress by decreasing ROS, reduces fat deposition, increases fatty acid uptake, improves insulin sensitivity; shows antioxidant and anti-inflammatory effects	Not reported	Not specified
Esan et al. [35]	Review article summarizing clinical trials and data on Colesevelam; includes patients with hypercholesterolemia and diabetes	To review the efficacy and safety of Colesevelam in lowering LDL-C and improving glycemic control	Colesevelam HCl monotherapy or in combination with other lipid-lowering or hypoglycemic agents	Lipid metabolism; glucose metabolism	Hypercholesterolemia, diabetes mellitus	LDL cholesterol levels, HbA1c, C-reactive protein levels	LDL-C reduced by 16–22% with monotherapy; additional 12–14% reduction when combined; HbA1c reduced by 0.5% (4 mmol/mol); CRP reduced by 16% monotherapy, 6% added to statins	Colesevelam effectively lowers LDL-C and HbA1c with fewer adverse effects than other BAS; useful as a third-line agent for hypercholesterolemia and glycemic control	Adverse effects on gut function; raises triglycerides; interferes with absorption of lipid-soluble drugs; limited use in GI disease or high triglycerides
Barbagallo et al. [36]	Narrative review; preclinical and clinical data on patients and animal models with type 2 diabetes	To review the effects of PDE5 inhibitors on cardiovascular complications in T2DM	Use of PDE5 inhibitors (sildenafil, tadalafil, vardenafil)	Cardiovascular and endothelial systems	Type 2 diabetes mellitus (T2DM), cardiovascular complications	Endothelial function (flow-mediated dilation), inflammatory cytokines, NO-cGMP-PKG pathway activity, cardiomyopathy, ischemia-reperfusion outcomes	Improvement in endothelial dysfunction; reduction in inflammatory cytokines; improved myocardial function; activation of NO-cGMP-PKG pathway	PDE5 inhibitors are beneficial in improving cardiovascular health in T2DM through multiple mechanisms, especially endothelial and anti-inflammatory effects	Not directly reported in this review (review-based synthesis)
Scammahorn et al. [37]	Narrative review; preclinical studies (animal models)	Explore the physiological and therapeutic roles of hydrogen sulfide (H₂S) in oxidative stress	H₂S supplementation via enzymatic (trans sulphuration, cysteine catabolism) and non-enzymatic pathways; potential therapeutic and procedural uses	Renal and cardiovascular	Oxidative stress-related conditions: cardiorenal diseases, aging-related disorders, procedural complications	Redox modulation, ROS scavenging, activation of antioxidant pathways (Keap1/Nrf2, NFκB, HIF-1α), potential organ protection	Not reported, Redox modulation, ROS scavenging, and activation of antioxidant pathways (Keap1/Nrf2, NFκB, HIF-1α) may provide potential organ protection.	H₂S shows strong therapeutic promise in renal and cardiovascular conditions, but clinical validation is needed	Not reported

Complications and challenges

Even though many benefits can come from using GLP-1 receptor agonists, Singh et al. indicated that bempedoic acid and FXR agonists in CRHM syndrome often lead to unwanted gastrointestinal, gout, liver, and itching side effects that make some patients less likely to keep using the treatment [25]. Li et al. showed that other challenges in caring for these patients involve broken communication among specialties, delaying doctors from making easy diagnoses, and a reluctance to change existing care routines [26]. Nikolaou et al. showed that using several drugs at once raises the risk of unpleasant interactions and decreases following the prescribed treatment, so attention to medications is necessary [21]. In addition, according to Vora et al., there are many important research gaps, mainly focusing on the gut-liver axis and microbiota-related therapies, because clinical trials have been limited in size, making it hard to develop proper guidelines and use these strategies in healthcare [22]. Therefore, we must establish cross-specialty approaches among cardiologists, nephrologists, hepatologists, endocrinologists, and primary care providers to address these disorders and improve patient outcomes.

Recommendation and future direction

The cardiovascular-renal-hepatic-metabolic syndrome requires an integrated care system involving cardiologists, nephrologists, hepatologists, endocrinologists, and primary care physicians. Early use of laboratory tests and advanced imaging is crucial for identifying minor heart issues. Scientists should focus on linking inflammation, mitochondrial dysfunction, and hormone disruption in various systems for new crossover treatments. Wearables and artificial intelligence (AI) in healthcare can help monitor patients and estimate diseases ahead of time. Better health policy development and patient learning can improve disease management. Longitudinal studies and clinical trials are essential for combining treatments. A global goal is to merge clinical guidelines for superior prevention, diagnosis, and treatment outcomes.

## Conclusions

Cardiovascular-renal-hepatic-metabolic syndrome is a new approach to understanding chronic diseases, focusing on the entire system rather than individual organs. Risk factors such as inflammation, stress, insulin resistance, and blood vessel defects worsen these conditions. This suggests the need for integrated, multidisciplinary solutions for diagnosis and management. Treating diseases at the molecular level and implementing lifestyle and personal medicine changes can prevent organ and health system decline. Adopting clinical guidelines and collaborating with experts from different specialties can improve patient outcomes and quality of life.
